# Adherence to First-Line Intravesical Bacillus Calmette-Guérin Therapy in the Context of Guideline Recommendations for US Patients With High-Risk Non-muscle Invasive Bladder Cancer

**DOI:** 10.36469/001c.124208

**Published:** 2024-10-28

**Authors:** Franklin D. Gaylis, Bruno Emond, Ameur M. Manceur, Anabelle Tardif-Samson, Laura Morrison, Dominic Pilon, Patrick Lefebvre, Lorie A. Ellis, Hiremagalur Balaji, Andrea Ireland

**Affiliations:** 1 1Genesis Healthcare Partners, San Diego, California, USA; 2 Analysis Group, Inc., Montréal, Québec, Canada; 3 3Janssen Scientific Affairs, LLC, Horsham, Pennsylvania, USA

**Keywords:** Bacillus Calmette-Guérin, non-muscle invasive bladder cancer, carcinoma in situ, treatment guidelines

## Abstract

**Background:** Bacillus Calmette-Guérin (BCG) can reduce recurrence and delay progression among patients with high-risk non–muscle invasive bladder cancer (NMIBC), but is associated with a substantial emotional, physical, and social burden. **Objectives:** This study evaluated the adequacy of first-line intravesical BCG treatment among high-risk NMIBC patients in the United States, including the subgroup with carcinoma in situ (CIS) of the bladder. **Methods:** Adults with high-risk NMIBC treated with BCG were selected from de-identified MarketScan® Commercial, Medicare, and Medicaid Databases (1/1/2010-2/28/2021). Adequacy of BCG induction and maintenance was evaluated from the first BCG claim until the end of the patient’s observation, using a previously published claims-based algorithm (induction: ≥5 instillations within 70 days; induction and maintenance: ≥7 instillations within 274 days of first instillation) and a definition based on the landmark Southwest Oncology Group (SWOG) trial (induction: ≥5 instillations without gaps >7 days; followed by ≥2 instillations at month 3, 6, and every 6 months thereafter). Proportions of patients with adequate BCG induction and maintenance were reported overall and compared between those with and without CIS. **Results:** Of 5803 high-risk NMIBC patients treated with first-line BCG (mean age, 67.3 years; 20.6% female), 930 (16.0%) had CIS. After first-line BCG, 56.6% received another treatment. Although 86.9% had adequate BCG induction based on the claims-based algorithm (SWOG, 73.6%), only 41.5% had adequate BCG induction and maintenance (SWOG, 1.6%). Similar trends were observed for patients with and without CIS, with higher adherence to guidelines for patients with CIS (adequate induction using claims-based algorithm: 90.3% vs 86.2%; adequate induction and maintenance: 50.8% vs 39.7%, all *P* < .001). A greater proportion of CIS patients than non-CIS patients had cystectomy (CIS, 14.4%, non-CIS, 8.5%; *P* < .001) after first-line BCG. **Discussion:** Among patients with NMIBC treated with first-line intravesical BCG, most received adequate BCG induction but less than half had adequate BCG maintenance. BCG treatment was also inadequate for patients with CIS, with only half of patients receiving adequate BCG maintenance and a higher proportion undergoing cystectomy following first-line BCG. **Conclusions:** Results emphasize the need for additional treatment options for patients with NMIBC.

## INTRODUCTION

Bladder cancer (BC) is the sixth most commonly diagnosed cancer in the United States (US), with 81 180 new cases reported in 2022,^1^ and is 4 times more prevalent in men than in women.^2^ Approximately 70% of BC diagnoses are non-muscle invasive (NMIBC).^3^ NMIBC has a 5-year survival rate of approximately 80%^4,5^ and is associated with substantial risk of recurrence and progression to muscle-invasive (MIBC) and metastatic disease (mBC).^6^ Carcinoma in situ (CIS) is an aggressive, high-risk subtype of NMIBC (5%-19% of cases)^7^ with an unfavorable prognosis.^4^

For intermediate-risk and newly diagnosed high-risk NMIBC, the American Urological Association/Society for Urologic Oncology (AUA/SUO) guidelines recommend adjuvant treatment with Bacillus Calmette-Guérin (BCG),^8^ an antiquated immunotherapy involving intravesical instillation of a live organism.^9^ BCG was approved by the US Food and Drug Administration (FDA) for the treatment of BC in 1990 based on Southwest Oncology Group (SWOG) trial results demonstrating significantly higher rates of recurrence-free survival (RFS) in patients with early-stage NMIBC and complete response in CIS patients treated with BCG vs intravesical doxorubicin chemotherapy.^10^ Both clinical trials and real-world studies have since shown that BCG treatment (induction plus maintenance) can reduce the risk of disease recurrence and delay progression compared with transurethral resection of the bladder tumor (TURBT) alone among patients with NMIBC.^11,12^

For high-risk patients with NMIBC, AUA/SUO guidelines advocate BCG induction consisting of a 6-week course of weekly instillations, followed by maintenance therapy for 3 years for patients who respond to BCG induction therapy.^8^ However, different definitions of BCG maintenance exist depending on the setting. In the SWOG trial, adequate BCG induction/maintenance schedule was 6 weeks of induction followed by 3-year maintenance,^13^ whereas in a real-world study, induction was at least 5 instillations within 70 days, with at least 7 instillations within 274 days of the first instillation as maintenance.^14^ Similarly, FDA guidelines recommend 6 weeks of weekly BCG instillations as induction followed by monthly instillations for 6 to 12 months.^15^ Irrespective of the applied definition, the frequent medical visits, voiding restrictions, and post-instillation cleaning protocols associated with BCG therapy^16^ impose a substantial emotional, physical, and social burden on patients and their families.^17^ This can in turn reduce adherence to therapy,^17^ resulting in inadequate treatment. Compounding this burden is the worldwide shortage of BCG due to manufacturing constraints since 2012.^18^ Moreover, nearly 80% of high-risk patients treated with BCG experience recurrence within 5 years,^6^ underscoring the substantial unmet therapeutic needs of this population.

Adherence to guidelines is crucial for achieving optimal outcomes, especially as BCG maintenance therapy has been shown to decrease tumor recurrence risk and prolong RFS over induction alone.^12,19^ To determine whether US patients with NMIBC are receiving adequate BCG treatment, this real-world study evaluated BCG treatment patterns in the context of clinical guideline recommendations in patients with high-risk NMIBC receiving BCG as first-line therapy, including a subgroup with CIS.

## METHODS

### Data Source

Data from the IBM® MarketScan® Commercial and Medicare Supplemental Databases (1/1/2010-2/28/2021) and Multi-State Medicaid Database (1/1/2010-12/31/2019) were used. The Commercial Database is an employer- and health plan–sourced database, with information on workers, their spouses, and dependents who are beneficiaries of private health insurance. The Medicare Supplemental Database comprises information on retirees with employer-paid Medicare Supplemental insurance. The Multi-State Medicaid database reflects the healthcare experience of approximately 7 million Medicaid enrollees from multiple states. The databases include information on enrollment, hospitalizations, prescription drugs, outpatient, and other services. Data were de-identified and complied with the patient requirements of the Health Insurance Portability and Accountability Act; therefore, no reviews by an institutional review board were required.

### Study Design

**Figure 1.**
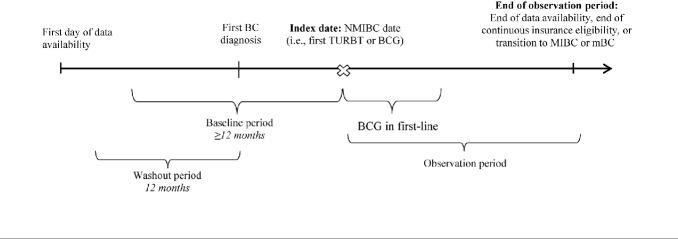
Study Design Scheme Abbreviations: BC, bladder cancer; BCG, Bacillus Calmette–Guérin; mBC, metastatic bladder cancer; MIBC, muscle-invasive bladder cancer; NMIBC, non–muscle-invasive bladder cancer; TURBT, transurethral resection of the bladder tumor.

A retrospective cohort study design was used (**Figure 1**). The index date (ie, date of NMIBC confirmation) was defined as the date of the first TURBT surgery or initiation of first-line BCG on or after the first BC diagnosis, whichever occurred first. The baseline period corresponded to the period at least 12 months before the index date. A washout period of at least 12 months of continuous eligibility before the first BC diagnosis was required to identify disease start.

The study period spanned from January 1, 2010, to February 28, 2021 (for Commercial and Medicare Supplemental patients), or December 31, 2019 (for Medicaid patients; ie, end of data availability). The index period (ie, period during which index dates could occur) spanned from January 1, 2011, to the end of data availability. The observation period spanned from the index date to the end of data availability, end of continuous insurance eligibility, or date associated with the NMIBC phase end (ie, transition to MIBC [ie, initiation of systemic antineoplastic therapy, radiotherapy, or cystectomy] or mBC diagnosis), whichever came first. To minimize the risk of survivor bias, no minimum observation period length was imposed.

For patients to be considered as initiating BCG in first line, BCG had to be the first intravesical therapy administered on or after the index date. All agents received within 42 days of BCG initiation were considered as part of the first-line regimen. Discontinuation of BCG was defined as switching to a new antineoplastic agent not included in the first-line regimen or having a treatment gap of more than 180 days. Switching to a new antineoplastic agent or re-starting BCG after a gap of more than 180 days triggered the start of a new line of therapy.

### Selection Criteria

**Figure 2.**
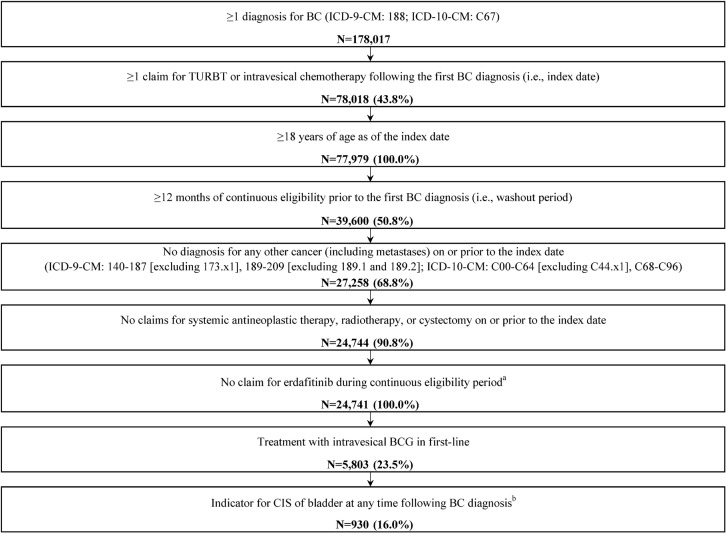
Sample Selection ^a^Approved for metastatic urothelial carcinoma with fibroblast growth factor receptor 3 (FGFR3) or FGFR2 gene alterations; Generic Product Identifier: 21 53 22 25 00. ^b^Patients with CIS were identified based on having at least two CIS diagnoses (ICD-9-CM: 233.7; ICD-10-CM: D09.0) between the first BC diagnosis and the end of the NMIBC phase (ie, earliest of the end of continuous health insurance eligibility, or the day before the first claim for systemic antineoplastic, cystectomy, radiotherapy, or metastatic disease). Abbreviations: BC, bladder cancer; BCG, Bacillus Calmette–Guérin; CIS, carcinoma in situ; ICD-9-CM/ICD-10-CM, *International Classification of Disease, Ninth/Tenth Revision, Clinical Modification*; NMIBC, non-muscle invasive bladder cancer; TURBT, transurethral resection of the bladder tumor.

Given the lack of an *International Classification of Diseases, Ninth/Tenth Revision, Clinical Modification* (ICD-9-CM/ICD-10-CM) diagnosis code for NMIBC, a claims-based algorithm was used to identify patients with NMIBC who received BCG as first-line therapy (**Figure 2**).

Patients were required to meet the following criteria:

- At least 1 diagnosis for BC (ICD-9-CM: 188; ICD-10-CM: C67) during the continuous eligibility period
- At least 1 claim for TURBT or intravesical therapy following the first BC diagnosis (ie, index date)
- Age 18 years or older at index
- At least 12 months of continuous eligibility before the first BC diagnosis (ie, washout period)
- No diagnosis of any other cancer (including metastases) on or before index (ICD-9-CM: 140-187 [excluding 173.x1] and 189-209 [excluding 189.1 and 189.2]; ICD-10-CM: C00-C64 [excluding C44.x1] and C68-C96)
- No claims for systemic antineoplastic therapy, radiotherapy, or cystectomy on or before index
- No claim for erdafitinib during continuous eligibility
- Treatment with intravesical BCG in first line

Patients with CIS were identified based on having at least 2 diagnoses for CIS of the bladder (ICD-9-CM: 233.7; ICD-10-CM: D09.0) between the first BC diagnosis and the end of the NMIBC phase (earliest of end of continuous health insurance eligibility, or the day before the first claim for systemic antineoplastic, cystectomy, radiotherapy, or metastatic disease).

### Study Measures

Patient demographic and clinical characteristics were described during the baseline period.

BCG treatment adequacy, per Williams et al^13^ and SWOG guidelines,^14^ was evaluated from the first BCG claim until the end of the observation period. Adequate induction and maintenance was defined by Williams et al as at least 5 BCG claims within 70 days and at least 7 BCG claims within 274 days, respectively, of the first BCG claim.^14^ SWOG guidelines defined adequate induction as at least 5 BCG claims without gaps of more than 7 days and adequate maintenance as: 1 year of maintenance (ie, ≥2 BCG claims at 3, 6, and 12 months) for patients with at least 12 months of follow-up; 2 years of maintenance (ie, ≥2 BCG claims at 3, 6, 12, 18, and 24 months) for patients with at least 24 months of follow-up; and 3 years of maintenance (ie, ≥2 BCG claims at 3, 6, 12, 18, 24, 30, and 36 months) for patients with at least 36 months of follow-up.^13^

Subsequent lines of therapy or any intravesical therapy following end of first-line BCG therapy (ie, last day of supply of first-line BCG) were also evaluated.

### Statistical Analysis

Means, SD, medians, and interquartile ranges were reported for continuous variables whereas frequencies and proportions were reported for categorical variables. A sunburst chart summarized treatment sequences as regimens received, stratified by line of therapy. Adequacy of BCG treatment and the proportion of patients having subsequent lines of therapy were compared between patients with and without CIS using *t* tests (continuous variables) and χ^2^ tests (categorical variables). *P* values <.05 indicate statistical significance, which reflects a difference in outcomes between these patients. All analyses were conducted using SAS Enterprise Guide software version 7.1 (SAS Institute).

## RESULTS

### Patient Characteristics

A total of 5803 NMIBC patients were treated with BCG in first line (mean [median] follow-up, 29.9 [24.1] months); 930 (16.0%) of these patients had documented CIS (**Table 1**). Overall, the population had a mean age of 67.3 years (range, 18-101 years), and was similar for patients with CIS (mean, 67.5 years) and patients without CIS (mean, 67.3 years; *P =* .753). A greater proportion of male patients was observed in the CIS cohort (83.4%) than in the non-CIS cohort (78.6%; *P <* .001). Compared with patients without CIS, patients with CIS had a higher proportion of urinary bladder catheterization (CIS, 7.3%, non-CIS, 5.0%; *P =* .005), urinary tract infections (CIS, 34.1%, non-CIS, 30.4%; *P =* .026), and kidney stones (CIS, 19.1%, non-CIS, 15.6%; *P* = .008).

**Table 1.** Demographic and Clinical Characteristics^a^

	**Overall First-Line BCG Population (N = 5803)**	**With CIS (N = 930)**	**Without CIS (N = 4873)**	***P* Value^b^**
Age, years, mean ± SD [Q1, median, Q3]	67.3 ± 11.4 [60.0, 66.0, 76.0]	67.5 ± 11.0 [60.0, 66.5, 75.0]	67.3 ± 11.4 [59.0, 66.0, 76.0]	.753
Sex, n (%)				
Female	1195 (20.6)	154 (16.6)	1041 (21.4)	<.001*
Male	4608 (79.4)	776 (83.4)	3832 (78.6)	<.001*
Region (among Commercial and Medicare only), n (%)				
South	1923 (33.9)	277 (29.8)	1646 (33.8)	.018*
North Central	1521 (26.8)	250 (26.9)	1271 (26.1)	.612
Northeast	1410 (24.9)	244 (26.2)	1166 (23.9)	.132
West	781 (13.8)	141 (15.2)	640 (13.1)	.097
Unknown	31 (0.5)	0 (0.0)	31 (0.6)	.015*
Year of index date, n (%)				
2011-2015	4251 (73.3)	726 (78.1)	3525 (72.3)	<.001*
2016-2020	1552 (26.7)	204 (21.9)	1348 (27.7)	<.001*
Payer, n (%)				
Commercial	2669 (46.0)	421 (45.3)	2248 (46.1)	.628
Medicare	2997 (51.6)	491 (52.8)	2506 (51.4)	.444
Medicaid	137 (2.4)	18 (1.9)	119 (2.4)	.351
Time from first BC diagnosis to index date, months, mean ± SD [Q1, median, Q3]	0.6 ± 2.8 [0.0, 0.1, 0.5]	0.7 ± 3.0 [0.0, 0.1, 0.6]	0.6 ± 2.7 [0.0, 0.1, 0.5]	.130
Prior BC-related procedures, n (%)				
Imaging	5562 (95.8)	899 (96.7)	4663 (95.7)	.172
Cystoscopy/cystourethroscopy	4345 (74.9)	714 (76.8)	3631 (74.5)	.145
Urinary bladder catheterization	314 (5.4)	68 (7.3)	246 (5.0)	.005*
Quan-Charlson Comorbidity Index, mean ± SD [Q1, median, Q3]	2.7 ± 2.2 [1.0, 2.0, 4.0]	2.7 ± 2.2 [1.0, 2.0, 4.0]	2.7 ± 2.2 [1.0, 2.0, 4.0]	.996
Other comorbidities, n (%)				
Urinary tract infection	1799 (31.0)	317 (34.1)	1482 (30.4)	.026*
Smoking	1204 (22.5)	200 (21.5)	1088 (22.3)	.581
Kidney stone	859 (16.1)	178 (19.1)	762 (15.6)	.008*

^a^Demographic and clinical characteristics were captured during the baseline period (≥12 months).

^b^*P* values comparing baseline characteristics between patients with and without CIS were calculated using t-tests for continuous variables and χ^2^ tests for categorical variables. An asterisk (*) denotes statistical significance at *P* < .05.

Abbreviations: BC, bladder cancer; BCG, Bacillus Calmette-Guérin; CIS, carcinoma in situ; Q1, first quartile; Q3, third quartile.

### Adherence to Treatment Guidelines

Most patients in the overall cohort (86.9%) completed BCG induction according to the method of Williams et al (CIS, 90.3%; non-CIS, 86.2%; *P <* .001), whereas only 41.5% of the overall cohort (CIS, 50.8%; non-CIS, 39.7%; *P <* .001) completed BCG maintenance (**Table 2**). Per SWOG guidelines, among patients with at least 12 months of follow-up from the first BCG claim, 73.6% completed BCG induction (CIS, 78.0%, non-CIS, 72.5%; *P =* .005), whereas only 1.6% also completed 1 year of maintenance (CIS, 1.6%; non-CIS, 1.6%; *P =* .980). Among patients with at least 24 months of follow-up, 75.0% completed BCG induction (CIS, 79.3%; non-CIS, 73.9%; *P =* .017), whereas only 0.3% also completed 2 years of maintenance (CIS, 0.2%; non-CIS, 0.4%; *P =* .592). Among patients with at least 36 months of follow-up, 76.1% completed BCG induction (CIS, 79.4%; non-CIS, 75.2%; *P =* .123) and no patients completed induction plus 3 years of maintenance.

### Treatment Patterns Following First-Line BCG

Most patients in the overall cohort (56.6%) had another treatment after first-line BCG (CIS, 70.0%; non-CIS, 54.0%; *P <* .001) (**Table 2**). A greater proportion of patients in the CIS cohort than the non-CIS cohort received any intravesical therapy (CIS, 57.6%; non-CIS, 52.7%; *P =* .024) and had cystectomy (CIS, 14.4%; non-CIS, 8.5%; *P <* .001) after first-line BCG.

**Table 2.** Treatment Patterns in NMIBC Patients

	**Overall First-Line BCG Population (N = 5803)**	**With CIS (N = 930)**	**Without CIS (N = 4873)**	***P* Value^a^**
Adequate BCG treatment, n (%)				
Per Williams et al (2021)				
Completed induction (≥5 BCG claims within 70 days of first BCG claim)	5040 (86.9)	840 (90.3)	4200 (86.2)	<.001*
Completed induction and maintenance ≥7 BCG claims within 274 days of first BCG claim)	2406 (41.5)	472 (50.8)	1934 (39.7)	<.001*
Per SWOG Guidelines (Lamm et al, 2000)				
Patients with ≥1 BCG claim beyond induction and ≥12 mo of follow-up from first BCG claim	3176 (54.7)	615 (66.1)	2561 (52.6)	<.001*
Completed induction (≥5 BCG claims) without gaps >7 days between claims	2336 (73.6)	480 (78.0)	1856 (72.5)	.005*
Completed induction (≥5 BCG claims) and 1 y maintenance (≥2 claims at 3, 6, and 12 mo)	52 (1.6)	10 (1.6)	42 (1.6)	.980
Patients with ≥1 BCG claim beyond induction and ≥24 mo of follow-up from first BCG claim	2287 (39.4)	459 (49.4)	1828 (37.5)	<.001*
Completed induction (≥5 BCG claims) without gaps >7 days between claims	1715 (75.0)	364 (79.3)	1351 (73.9)	.017*
Completed induction (≥5 BCG claims) and 2 y maintenance (≥2 claims at 3, 6, 12, 18, and 24 mo)	8 (0.3)	1 (0.2)	7 (0.4)	.592
Patients with ≥1 BCG claim beyond induction and ≥36 mo of follow-up from first BCG claim	1545 (26.6)	315 (33.9)	1230 (25.2)	<.001*
Completed induction (≥5 BCG claims) without gaps >7 days between claims	1175 (76.1)	250 (79.4)	925 (75.2)	.123
Completed induction (≥5 BCG claims) and 3 y maintenance (≥2 claims at 3, 6, 12, 18, 24, 30, and 36 mo)	0 (0.0)	0 (0.0)	0 (0.0)	–
Any treatment following the end of first-line BCG,^b^ n (%)	3283 (56.6)	651 (70.0)	2632 (54.0)	<.001*
Any intravesical therapy	1761 (53.6)	375 (57.6)	1386 (52.7)	.024*
Any systemic chemotherapy	701 (21.4)	133 (20.4)	568 (21.6)	.521
TURBT	2471 (75.3)	510 (78.3)	1961 (74.5)	.042*
Radiotherapy	258 (7.9)	33 (5.1)	225 (8.5)	.003*
External beam radiotherapy	216 (6.6)	26 (4.0)	190 (7.2)	.003*
Trimodal therapy (systemic chemotherapy, TURBT, and radiotherapy at any time)	126 (3.8)	16 (2.5)	110 (4.2)	.041*
Cystectomy	317 (9.7)	94 (14.4)	223 (8.5)	<.001*
Hospice stay	152 (4.6)	27 (4.1)	125 (4.7)	.513

^a^*P* values comparing treatment patterns between patients with and without CIS were calculated using χ^2^ tests for categorical variables. An asterisk (*) denotes statistical significance at *P* < .05.

^b^Treatment patterns were described from the end of active first-line BCG therapy (ie, last day of supply of BCG in first line) until end of data availability for a patient (ie, earliest of end of continuous insurance eligibility or end of data availability or censoring date associated with transition to muscle-invasive bladder cancer [ie, initiation of systemic antineoplastic therapy, radiotherapy, or cystectomy] or diagnosis of metastatic bladder cancer).

Abbreviations: BCG, Bacillus Calmette-Guérin; CIS, carcinoma in situ; NMIBC, non-muscle invasive bladder cancer; SWOG, South Western Oncology Group; TURBT, transurethral resection of the bladder tumor.

**Figure 3.**
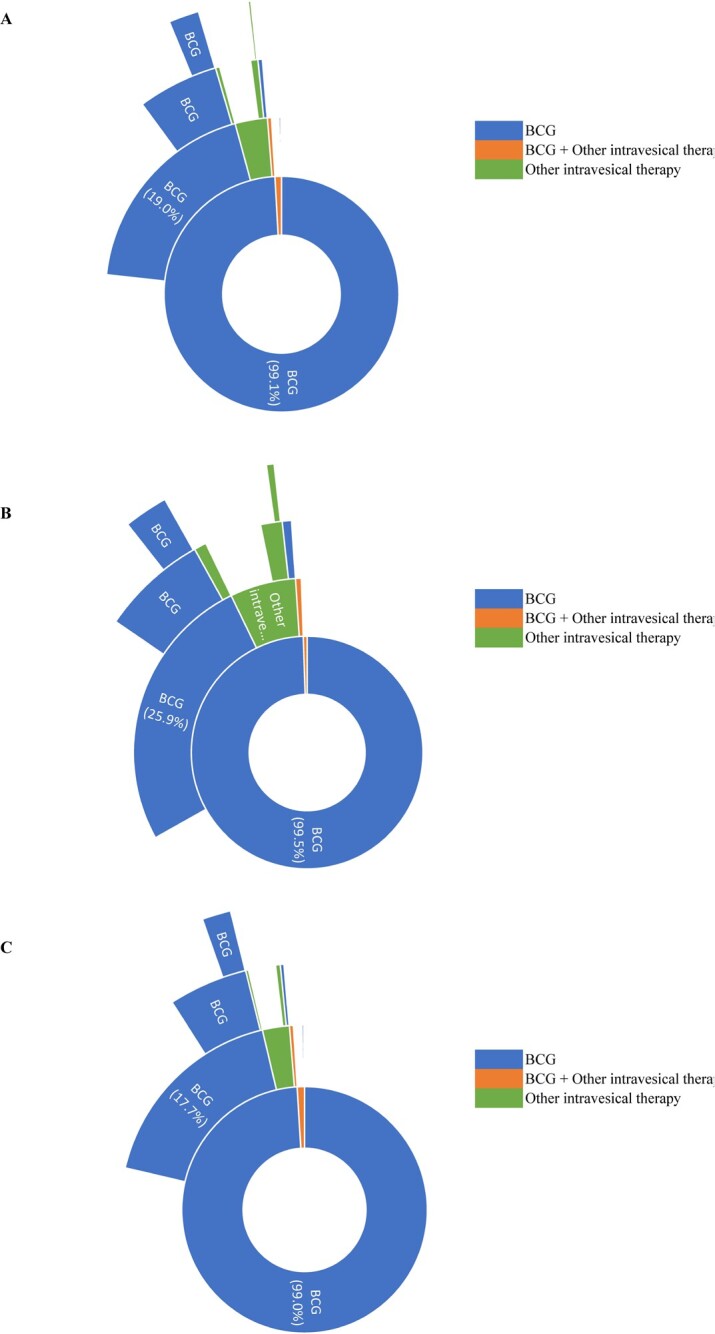
Treatment Sequences in (**A**) All Patients, (**B**) Patients With CIS, and (**C**) Patients Without CIS Treated With BCG in First Line of Therapy^a,b,c,d^ ^a^Each concentric ring represents 1 line of therapy, with the innermost circle representing the first line and the outermost ring representing the fourth line. ^b^Only lines initiated ≥42 days from the end of the NMIBC phase were included in the analysis to ensure that all agents in the line of therapy regimen were identified. Patients with >90 days between the last line’s last day of treatment (>180 days if the last line contained BCG) and end of the observation period were considered to have discontinued. Patients with <90 days between the last line’s last day of treatment (<180 days if the last line contained BCG) and end of the observation period were considered censored. ^c^To improve visualization in later lines of therapy, only categories with >2 patients are shown. ^d^Other intravesical therapies included mitomycin, gemcitabine, docetaxel, and valrubicin. Abbreviations: BCG, Bacillus Calmette–Guérin; CIS, carcinoma in situ; NMIBC, non–muscle-invasive bladder cancer.

More than three-quarters of patients treated with BCG in first-line therapy discontinued treatment without a subsequent line of therapy (**Figure 3A**). BCG was the predominant treatment across lines of therapy; 2.5% of all patients switched to a different intravesical therapy either alone or in combination with BCG following first-line BCG treatment. A larger proportion of patients in the CIS cohort compared with patients in the non-CIS cohort had BCG retreatment in the second line of therapy (CIS, 25.9%; non-CIS, 17.7%; *P <* .001), with BCG remaining the predominant treatment across lines of therapy (**Figure 3B and 3C**).

## DISCUSSION

This real-world study evaluated the adequacy of BCG treatment patterns in a population of US patients with NMIBC, including those with CIS, following initiation of first-line therapy with BCG. The main finding of this study was the relatively poor adherence to guideline-recommended BCG maintenance, which highlights the need for interventions to improve adherence and patient education. In addition, the findings from this study highlight the need for alternative therapies to BCG for patients with NMIBC, especially those with CIS and/or intermediate- to high-risk, who may require more aggressive disease management.

Although most patients with NMIBC in this study received adequate BCG induction, regardless of the applied definition of induction,^13,14^ fewer than half had adequate BCG maintenance, including patients at the highest risk of progression (CIS subgroup). After discontinuing BCG treatment, most patients had no additional treatment and nearly 1 in 10 elected to undergo cystectomy.

The various definitions of adequate BCG induction and maintenance represent a clinical challenge in NMIBC disease management. Besides those used in the current study,^13,14^ the FDA defines adequate BCG therapy as 6 weeks of weekly BCG instillations (≥5 of 6 doses) as induction followed by monthly instillations for 6 to 12 months (≥2 of 3 doses of maintenance or 2 of 6 doses of a second induction course).^15^ The International Bladder Cancer Group has a similar definition for clinical trials, imposing at least 1 maintenance course (2 of 3 instillations) within a 6-month period.^20^ In the SWOG trial, 16% of patients completed the 3-year maintenance course^13^; however, in the current study, no patients completed induction and 3 years of maintenance per SWOG guidelines. Using a definition adapted for real-world data, 37% of veterans in the Williams et al study received adequate BCG maintenance,^14^ which is comparable to the 42% in the overall NMIBC cohort in the current study. Similarly, prior research across 9 studies of 4452 patients with high-risk NMIBC found a 32.5% rate of compliance with adjuvant intravesical BCG.^21^ Irrespective of the adopted definition, results from this study and others indicate that NMIBC patients receive suboptimal BCG treatment (particularly maintenance).^22^

Multiple factors may contribute to inadequate BCG treatment in patients with NMIBC. The procedure itself—which includes multiple visits to the physician’s office, the need to rotate the bladder, a specific voiding protocol, and a decontamination regime—is arduous.^16^ Additionally, a systematic literature review found that urinary adverse events, such as frequent and painful urination and hematuria, were reported in up to 97% of patients and were major reasons for BCG discontinuation.^17^ The constraint on BCG supply has also been shown to negatively impact treatment compliance. For instance, in a single-center study conducted in the United Kingdom, 30% of patients with intermediate- or high-risk NMIBC were noncompliant to BCG treatment due to the shortage (compliance defined as completing 12 doses of BCG within the first year following diagnosis).^23^ The limited availability of BCG has also prompted modifications to the BCG regimen, including AUA recommendations to use one-third of a BCG dose for induction and limit maintenance to 1 year (although patients with high-risk NMIBC or CIS are prioritized for receipt of full-strength BCG, if feasible).^8,24^ However, a systematic review of real-world studies reported shorter RFS for patients without vs with BCG maintenance.^19^ Thus, failure to adhere to guideline-recommended treatment regimens can lead to suboptimal patient outcomes. While not evaluated in this study, patient-centered approaches that tailor treatment plans to the individual patient, involve caregivers, and increase engagement may have a beneficial effect on clinical outcomes and warrant further research.^25^

Besides receiving inadequate treatment, many patients become unresponsive to BCG treatment in first-line (44%) or second-line (22%) therapy^26^ yet have few alternative treatment options. This is particularly true for CIS, a noninvasive but aggressive form of NMIBC with a high risk of progression and metastasis.^27^ In the present study, a higher proportion of patients in the CIS cohort compared with the non-CIS cohort received another treatment following first-line BCG (70% vs 54%) including cystectomy (14% vs 9%), suggesting their disease was not controlled after first-line treatment. These results suggest there is an important need for patients with CIS to adhere to treatment guidelines and have alternative therapeutic options.

Even after BCG therapy, recurrence and progression rates remained high, especially for high-risk disease (5-year RFS, 23%; 5-year progression-free survival, 54%).^6^ As such, follow-up monitoring is recommended by NMIBC treatment guidelines.^8^ For low-risk patients, AUA/SUO guidelines for BC recommend a follow-up cystoscopy within 3 to 4 months of TURBT; if findings are negative, a cystoscopy should be performed 6 to 9 months later and yearly for 5 years.^8^ For patients with high-risk NMIBC, surveillance through follow-up cytology/cystoscopy every 3 to 4 months for 2 years after TURBT and BCG treatment, and at longer intervals thereafter, is recommended.^8^ The European Association of Urology (EAU) and Canadian Urological Association (CUA) recommend a cystoscopy 3 months after TURBT for both low- and high-risk NMIBC.^28,29^ According to the EAU, surveillance cystoscopies should be performed for 5 years (low-risk) or life-long (high-risk).^29^ The CUA specifies annual cystoscopy for 5 years after TURBT (low-risk), and cytology/cystoscopy every 3 to 4 months for 2 years, every 6 months for the next 2 years, and annually thereafter (high-risk).^28^ In addition to surveillance, implementing screening programs^30^ and educating physicians on risk factors for the development of BC, including tobacco smoking and exposure to toxins^2^ as well as chronic inflammation,^31^ may lead to earlier detection.

This study had certain limitations. First, the use of claims data did not allow for precise identification of patients with NMIBC or the actual date of the first BC diagnosis, which was approximated using a 12-month washout period. This may have resulted in patient misclassification, but this was mitigated by incorporating as much information as possible from the claims data into the claims-based algorithm. Second, CIS was identified based on the presence of a diagnosis code for CIS of the bladder at any time after the index date, but it is not known if the patient had CIS on the index date. Thus, given the absence of test results in the database, the presence of a diagnosis code for CIS at any time during the NMIBC phase served as an indication that the patient had CIS of the bladder. Third, as this was an analysis of claims data, there was a lack of clinical information on oncologic parameters such as tumor grade, disease stage, disease status (ie, remission or recurrence/progression); therefore, some patients may have been misclassified and treatment guidelines may not have applied to all patients analyzed. Similarly, reasons for treatment discontinuation (eg, supply shortage, intolerance, or declining patient compliance) were not available. As a result, it was not possible to separate results between the different reasons for discontinuation. Fourth, there was a risk of identifying patients who used antineoplastic agents for cancers other than BC, although this was minimized by excluding patients with other cancers prior to BC diagnosis. Fifth, the findings may not be generalizable to patients without health insurance, and reimbursement policies may differ across plans. Sixth, coding inaccuracies in claims data may have resulted in misidentification. However, this was expected to affect all patients equally. Finally, given the descriptive nature of the study, no adjustments were made for potential confounders.

## CONCLUSIONS

In this real-world study of patients with NMIBC treated with first-line intravesical BCG, most received adequate BCG induction, but less than half had adequate BCG maintenance. BCG treatment was also inadequate for patients with CIS, with only half of patients receiving adequate BCG maintenance and a higher proportion undergoing cystectomy following first-line BCG. Considering low adherence to guidelines, supply constraints for BCG, and potential tolerability and lifestyle demands placed on patients undergoing BCG therapy, the results of this study underscore a need for additional treatment options for patients with NMIBC. Moreover, this study highlights the importance of promoting adherence to treatment guidelines and long-term surveillance to achieve optimal clinical outcomes.

### Disclosures

F.D.G. is an employee of Unio Health Partners and reports the following relationships: Janssen Pharmaceuticals (consultant), Labcorp (consultant), Fortrea (consultant), and Invitae (consultant). B.E., A.M.M., A.T.-S., L.M., D.P., and P.L. are employees of Analysis Group, Inc., a consulting firm that received research funding from Janssen Scientific Affairs, LLC, to conduct this study. L.A.E., H.B., and A.I. are employees of Janssen Scientific Affairs, LLC, and stockholders of Johnson & Johnson.

### Data Availability Statement

Data that support the findings of this study were used under license from IBM MarketScan®. Restrictions apply to the availability of these data, which are not publicly available and cannot be shared. The data are available through request made directly to the data vendor, subject to the data vendor’s requirements for data access.

### Presentations

Part of the material in this manuscript was presented at ASCO Genitourinary Cancers Symposium, February 16-18, 2023, San Francisco, California, and online.
